# Heat shock protein 27 plays a protective role in thoracic aortic dissection by promoting cell proliferation and inhibiting apoptosis

**DOI:** 10.1186/s11658-017-0056-y

**Published:** 2017-11-28

**Authors:** Sili Zou, Mingfang Liao, Junlin Yang, Tong Huang, Mark Green, Jianjin Wu, Lefeng Qu

**Affiliations:** 1 Department of Vascular and Endovascular Surgery, Changzheng Hospital, the Second Military Medical University, 415 Fengyang Road, Shanghai, People’s Republic of China; 2 DICAT Biomedical Computation Centre, Vancouver, BC Canada

**Keywords:** Aortic dissection, Heat shock protein 27, Oxidative stress, Thoracic aortic dissection, Vascular smooth muscle cells

## Abstract

**Background:**

Thoracic aortic dissection (TAD) is one of the most severe aortic diseases. The study aimed to explore the potential role of heat shock protein 27 (HSP27) in the pathogenesis of TAD using an in vitro model of oxidative stress in vascular smooth muscle cells (VSMCs).

**Methods:**

HSP27 was analyzed in aortic surgical specimens from 12 patients with TAD and 8 healthy controls. A lentiviral vector was used to overexpress HSP27 in rat aortic VSMCs. Cell proliferation and apoptosis were measured under oxidative stress induced by H_2_O_2_.

**Results:**

HSP27 expression was significantly higher in aortic tissue from patients with TAD and VSMCs in the aortic media were the main cell type producing HSP27. Elevated oxidative stress was also detected in the TAD samples. Overexpression of HSP27 significantly attenuated H_2_O_2_-induced inhibition of cell proliferation. Furthermore, HSP27 was found to decrease H_2_O_2_-induced cell apoptosis and oxidative stress.

**Conclusions:**

These results suggest that HSP27 expression promotes VSMC viability, suppresses cell apoptosis, and confers protection against oxidative stress in TAD.

## Background

Thoracic aortic dissection (TAD) is one of the most severe aortic diseases. Its onset involves separation of the layers of aortic wall and entry of blood to the vessel wall through this intimal tear [1]. If the tear extends into the aortic wall media, pulsatile blood can tear it apart along the length of the aorta [2].

The aortic wall is constantly subjected to biological insults and hemodynamic stress, leading to aortic wall degeneration with the possibility of dissection or aneurysm [3]. The structural changes of the aortic wall over time include fragmentation of elastic fibers, focal or zonal necrosis of the media, and the transition of smooth muscle cells (SMCs) from a contractile to asynthetic phenotype [4].

Oxidative stress is an important factor causing tissue injury, but the role of oxidative stress in TAD is poorly understood. Heat shock proteins (HSPs), also known as cell stress proteins, are chaperone proteins mainly expressed in the cytoplasm. They respond to heat shock or cellular stress caused by a variety of factors, such as toxic compounds, ischemia or free radicals. They bind temporarily to other molecules in the cell and assist in the maintenance of their non-covalent structures [5]. Under normal conditions, HSPs are present in small amounts, but their levels increase greatly under cellular stress conditions. They participate in the digestion and recycling of damaged proteins, and may assist in the correct folding of newly synthesized proteins [6].

Many studies have attempted to understand the pathogenesis of TAD. They did provide important insights into the disease mechanisms, but most of them focused on genetic heterogeneity, clinical pathology and the hemodynamics of TAD [7]. Comparative proteomics studies of diseased vs. normal tissue have been used to investigate coronary and carotid atherosclerosis and the SMC secretome [8], but few such studies have been undertaken for TAD, which is a non-atherosclerotic vascular disease.

One of our previous proteomic studies showed that HSP27, a member of the small HSP family [9], is differentially expressed in the aortic media of patients with TAD compared with controls [10], but whether and how HSP27 plays a role in TAD is unknown. Desai et al. reported that as an ubiquitin-binding protein, HSP27 is associated with hydrogen peroxide-inducible clone 5 (Hic-5) and regulates the reactive oxygen species-generating enzyme NADPH oxidase 4 (Nox4), and thus plays an important role in myofibroblast differentiation and senescence [11, 12]. This led us to consider the role of HSP27 in vascular SMCs (VSMCs), the most important component of the medial layer of aortic wall.

The aim of this study is to explore the potential roles of HSP27 in the pathogenesis of TAD using VSMCs, and to compare the oxidative stress levels and HSP27 expression in the aortic tissue of patients with TAD and control subjects. This study could provide more information about the role of oxidative stress in TAD, improving the understanding of the pathogenesis of TAD.

## Methods

### Patients and samples

The subjects were patients who underwent elective surgical repair of ascending TAD (*n* = 12). Exclusion criteria were: 1) bicuspid aortic valve (BAV); 2) heritable connective tissue disease (e.g., Marfan syndrome); 3) TAD related to trauma; 4) aortitis; 5) infection; or 6) first-degree relatives who had aortic aneurysm or dissection.

The study protocol was approved by the ethics committee of Changzheng Hospital. All patients gave a written informed consent.

Aortic samples were obtained during surgical repair of TAD. The samples were from the diseased part of the ascending aorta. Tissues were resected and washed in iced normal saline. Adherent clots and the outer membrane were removed. The tissues were then divided into pieces of 0.8 × 1.0 cm and stored in liquid nitrogen. Control ascending thoracic aortic tissue was collected from age-matched cadaver donors without aortic aneurysm, dissection, coarctation or previous aortic repair.

A detailed description of the aortic samples is presented in Table 1. All the samples were prepared in parallel. Briefly, media tissues were cut into small pieces and stored at −80 °C until protein extraction. Some tissues that were also collected from the diseased part of the ascending aorta were paraffin imbedded for immunohistochemistry validation using monoclonal antibodies against the SMC marker α-smooth muscle actin and the endothelial cell marker CD-31. Staining with hematoxylin and eosin or Victoria blue for elastic and collagen fibers respectively were done for all tissue samples using standard procedures.

**Table 1:** Characteristics of the patients and controls

Characteristics	Controls	TAD patients	*p*
	(*n* = 8)	(*n* = 12)	
Age (y)	40.9 ± 6.7	47.4 ± 9.2	0.4
Men	8 (100%)	12 (100%)	1
History of smoking	5 (62.5%)	6 (50%)	0.7
Hypertension	0 (0%)	9 (75%)	0.001
Atherosclerosis	3 (37.5%)	7 (58.3%)	0.7

Age and aortic diameter were compared using Student’s t-test. All other variables were compared using Fisher’s exact test. *TAD* thoracic aortic dissection

### Aortic sample preparation

Frozen aortic tissues (10 mg) were ground using a grinding tube with a matching pestle in an ice bath. The resulting powders were homogenized in 100 ml of lysis buffer (9 mol/l of urea, 4% of CHAPS, 65 mmol/l of dithiothreitol, and 1 mmol/l of phenylmethylsulfonyl fluoride) at 4 °C for 5 min, and then centrifuged at 4 °C at 14,000 rpm for 1 h. The supernatant was collected, and protein concentrations were quantified using the bicinchoninic acid Protein Assay Kit (Bio-Rad). Supernatant samples were divided into 50 ml aliquots and stored at −80 °C.

### Western blot

Proteins were extracted from the aortic media specimens with a commercial lysis buffer (CelLytic, Sigma-Aldrich Corporation). Western blot was performed with standard procedures. A mouse anti-HSP27 monoclonal antibody (1:1000, Cell Signaling Technology) and a horseradish peroxidase-labeled secondary antibody (1:5000, Santa Cruz Biotechnology) were used. The blots were quantified using the Image J software (National Institutes of Health).

### Oxidative stress assessment of aortic tissue

SOD activity, lipid peroxidation and catalase activity were measured to assess the oxidative stress of aortic tissue. A commercially available kit (Jiancheng Bioengineering Institute) was used to estimate SOD activity, which was based on the generation of superoxide radicals produced by xanthine and xanthine oxidase, which react with nitro blue tetrazolium (NTB) to form formazan dye. SOD activity was then measured at 550 nm by the degree of inhibition of this reaction. To distinguish the cyanide-sensitive isoenzyme copper-zinc SOD (Cu/Zn-SOD) and extracellular SOD (EC-SOD) from the cyanide-resistant manganese SOD (Mn-SOD), 3 mmol/l cyanide was used. One unit in the assay is defined as the activity that brings about a decay in O_2_ concentration at a rate of 0.1/s in 3 ml of buffer. The results are expressed as U/mg protein.

Lipid peroxidation was assessed by measuring the levels of malondialdehyde using a commercially available kit (Jiancheng Bioengineering Institute). Malondialdehyde content was determined based on the reaction of malondialdehyde with thiobarbituric acid at 90–100 °C. The absorbance of the supernatant was measured at 532 nm. Malondialdehyde concentrations were expressed as nmol/ml.

Catalase activity was determined by monitoring the breakdown of hydrogen peroxide catalyzed by catalase, using a commercially available kit (Jiancheng Bioengineering Institute). The reaction rate is determined by monitoring the decrease in the absorbance of a substrate of H_2_O_2_ at 520 nm. The results are expressed as U/mg protein.

### Immunohistochemistry and double immunofluorescence staining

Formalin-fixed, paraffin-embedded aortic sections were deparaffinized and rehydrated before antigen retrieval. The sections were incubated with mouse anti-HSP27 monoclonal antibody (1:50, Cell Signaling) at 4 °C overnight, and then incubated with peroxide-conjugated anti-mouse IgG secondary antibody and stained with 3, 3-diaminobenzidine using the Vectastain ABC kit (Vector Laboratories). For double immunofluorescence staining, the sections were incubated with mouse anti-HSP27 antibody (1:50, Cell Signaling) and rabbit anti-SM22α antibody (1:200, Abcam) at 4 °C overnight. Sections treated only with normal IgG were used as negative controls. Alexa Fluor 488- and Alexa Fluor 568-conjugated secondary antibodies (Invitrogen) were used.

### Cell culture

A10 rat aortic SMCs (ATCC CRL 1476; American Type Culture Collection) were grown in high-glucose Dulbecco’s modified Eagle’s medium (DMEM; GIBCO, Invitrogen Inc.) containing 10% fetal bovine serum (FBS; GIBCO, Invitrogen Inc.) in a humidified incubator with 5% CO_2_ at 37 °C.

### Lentiviral vector

*Hspb1* (Gen Bank: M86389.1), the gene for rat HSP27, was synthesized and assembled into the MD-18 T vector (Takara), then transfected into DH5α competent cells (Invitrogen Inc.). PCR was performed to amplify the correctly synthesized *Hspb1* (primer pair: F 5′-CGCGGATCCGCCACCATGACCGAGCGCCGC -3′; R 5′- CTAGCTAGCCTACTTGGCTCCAGACTGTTCCGACTCT-3′). The PCR product was loaded into the lentiviral vector pLenti6.3-MCS-IRES2-EGFP (Invitrogen Inc.). Infectious lentiviral particles containing the lentiviral vector pLenti6.3-MCS-IRES2-EGFP with *Hspb1* were produced as previously described [13]. Briefly, 293 T cells were plated on 10-cm dishes at a density of 6 × 10^6^ cells/plate, cultured overnight, and then co-transfected with the pLenti6.3-Hsp27-IRES2-EGFP construct (LVUT^Hspb1^) and a packaging mix (Invitrogen Inc.) with Lipofectamine 2000 (Invitrogen Inc.) in Opti-MEM (GIBCO, Invitrogen Inc.). Approximately 6 h later, the culture medium was replaced with DMEM containing 10% FBS and further incubated for 48 h. This medium was then collected and filtered through a 0.45-μm filter to remove any cellular debris. Viral stocks were stored at −80 °C until further use.

### Overexpression of HSP27

A10 cells were cultured on 6-well plates at a density of 1.5 × 10^5^ cells/well overnight. The medium was replaced with fresh growth medium containing 8 μg/ml of polybrene (Invitrogen Inc.) and LVUT^Hspb1^ or Lenti6.3-EGFP empty vector control (LVUT^EGFP^) infectious viral particles. The density of infectious viral particles was based on a multiplicity of infection (MOI) equal to 100. A10 cells without viral particles were prepared as controls. Forty-eight h after infection, 5 μg/ml of blasticidin (Invitrogen Inc.) was added to the medium. GFP expression of the cells was evaluated every day, and growth medium containing 5 μg/ml of blasticidin was changed every 2–3 days. When all the cells in the control well were dead, the A10 cells infected with LVUT^Hspb1^ or LVUT^EGFP^ were expanded, and the medium was replaced with fresh growth medium. Overexpression of HSP27 was assessed using western blot and quantitative PCR.

### Cell proliferation assay

Non-infected A10 cells, A10 cells infected with LVUT^EGFP^, and A10 cells infected with LVUT^Hspb1^ were sub-cultured in 96-well plates at a density of 1 × 10^4^ cells/well for 24 h. Then, the medium was replaced by DMEM without FBS to arrest growth for 24 h. Confluent growth-arrested cells were treated with 10% FBS with or without 2 mmol/l of H_2_O_2_ for 2 h at 37 °C. A cell proliferation assay was performed using the Cell Proliferation MTT Assay Kit (Cayman Chemical) according to the manufacturer’s instructions. The H_2_O_2_-induced inhibition rate of proliferation for each group was compared.

### Cell apoptosis

Non-infected A10 cells, A10 cells infected with LVUT^EGFP^, and A10 cells infected with LVUT^Hspb1^ were subcultured in 6-well plates at a density of 5 × 10^5^ cells/well for 24 h. Then, the medium was replaced by DMEM without FBS to arrest growth for 24 h. Confluent growth-arrested cells were treated with 10% FBS with 800 μmol/l of H_2_O_2_ for 24 h at 37 °C. Cell apoptosis was analyzed using the Annexin V-PE/7-AAD Apoptosis Detection Kit (KeyGen Biotech Co.) according to the manufacturer’s instructions. Briefly, cells were resuspended in the binding buffer at a density of 1 × 10^6^ cells/ml and successively incubated at 4 °C with 5 ml of Annexin V-PE/7-AAD for 15 min and 10 ml of propidium iodide (PI) for 5 min. Labeled cells were then detected using a fluorescence-activated cell sorter (FACS; BD Biosciences).

### Oxidative stress

The steps of treatment were the same as for the cell apoptosis experiment. Total superoxide dismutase (SOD) activity and lipid peroxidation were tested to assess oxidative stress. SOD activity was determined using a total SOD assay kit with WST-1 (Beyotime Institute of Biotechnology). The absorbance was measured at 450 nm, and the result was expressed as U/mg protein. Lipid peroxidation was determined by measuring the levels of malondialdehyde using a commercially available kit (Jiancheng Bioengineering Institute). Malondialdehyde content was determined based on the reaction of malondialdehyde with thiobarbituric acid at 90–100 °C. The absorbance of the supernatant was measured at 532 nm, and the result was expressed as nmol/mg protein.

### Statistical analysis

All experiments were repeated four times. Data are reported as means ± SD, compared using the Student’s t-test (between two groups) or one-way analysis of variance (among three or more groups). *P* < 0.05 was considered statistically significant. All statistical analyses were performed using SPSS 13.0 (SPSS Inc.).

## Results

### HSP27 levels were higher in the aortic wall of patients with TAD and was mainly produced by VSMCs

**Fig. 1:**
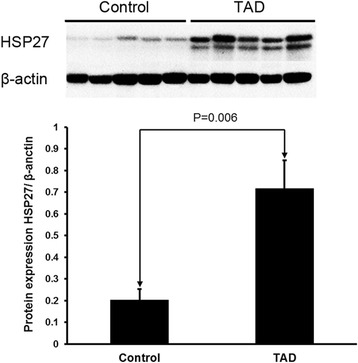
Western blots were performed using the protein lysate from aortic tissues. HSP27 protein levels were significantly increased in the aortic wall of TAD patients compared with controls (*p* = 0.006). β-actin was used as loading control protein. Control, *n* = 8; TAD, *n* = 12

To confirm that HSP27 expression is altered in TAD, western blots were performed using the protein lysate from aortic tissues. HSP27 protein levels were significantly increased in the aortic wall of patients with TAD compared with the controls (*p* = 0.006; Fig. 1).

**Fig. 2:**
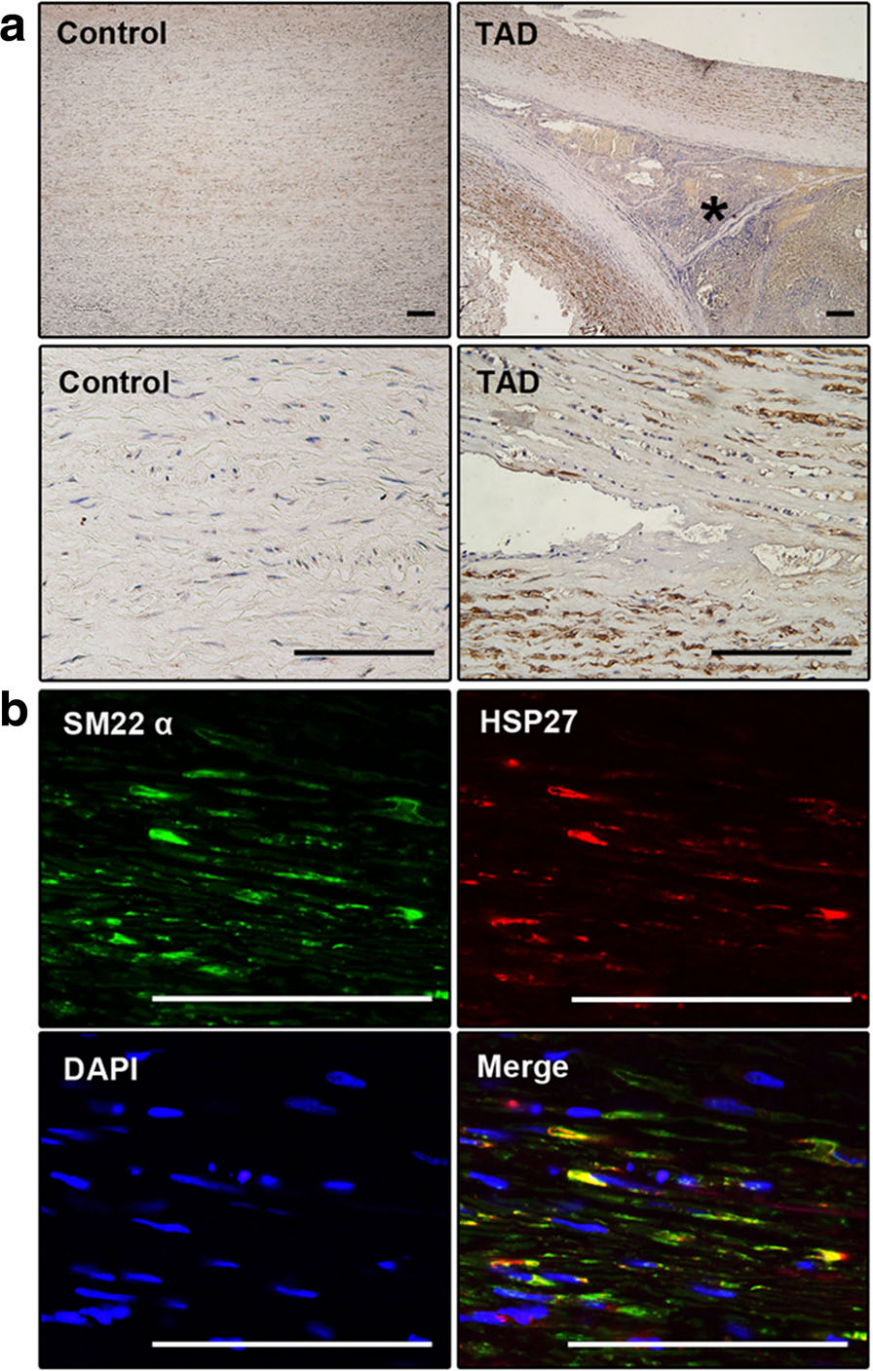
Immunohistochemical analysis of HSP27 in the aortic samples of both controls and patients. **a** – HSP27 expression was higher in the aortic media of patients with TAD than in that of controls. HSP27-negative bands are observed along the tear in the aortic media of patients with TAD. * indicates false lumen. Scale bar = 100 μm. **b** – Double immunofluorescence staining showing colocalization of HSP27 and SM22α in the aortic media of TAD patients. Scale bar = 100 μm

Immunohistochemistry showed that HSP27 was expressed in the aortic samples of both controls and patients with TAD, but that its level was significantly higher for patients with TAD. However, in the low-power field of view, HSP27-negative bands were observed along the tear in the aortic media of TAD patients, suggesting failure to produce HSP27 along the torn layer (Fig. 2a).

To determine which cells produce HSP27, double immunofluorescence staining of HSP27 and SM22α (a marker of SMCs) was carried out on the aortic samples of patients with TAD. A high proportion of co-localization of HSP27 and SM22α confirmed that VSMCs in the aortic media are the main cell type producing HSP27 (Fig. 2b).

### Oxidative stress was increased in the aortic wall of patients with TAD

**Fig. 3:**
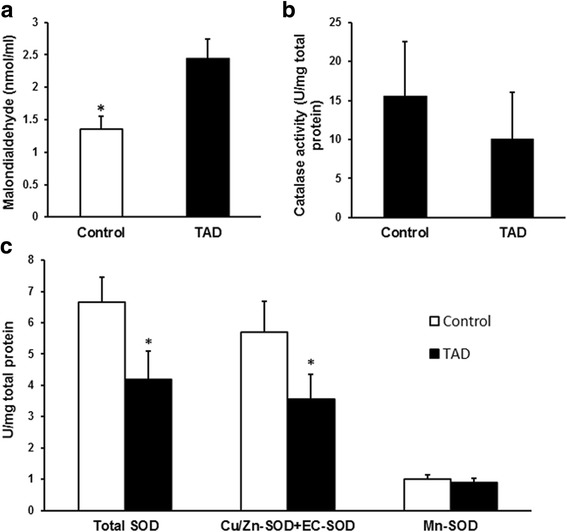
Malondialdehyde levels and catalase and SOD activities in TAD samples. **a** – The levels of malondialdehyde in the aortic media were significantly higher in the TAD group (*p < 0.05). **b** – Catalase activity was lower in the TAD group, but the difference was not statistically significant. **c** – Total SOD activity and activities of Cu/Zn-SOD plus EC-SOD had both decreased in the TAD group compared with the control group, but no significant differences in Mn-SOD activity were found between groups

EC-SOD expression levels had decreased significantly in the aortic wall of patients with TAD, suggesting an impaired antioxidative mechanism in TAD tissue. The levels of malondialdehyde, a product of lipid peroxidation, were consistently significantly higher in aortic media homogenates in the TAD group compared with controls (Fig. 3a, *p* < 0.05). No significant differences in catalase activity or Mn-SOD activity were found between groups, despite the decreased tendency in TAD group (Fig. 3b and c). Total SOD activity had decreased in the TAD group compared with the controls. The activity of cyanide-sensitive isoenzymes SOD (Cu/Zn-SOD plus EC-SOD) had significantly decreased in the TAD group compared with the controls (Fig. 3c).

### Overexpression of HSP27 in LVUT^Hspb1^-infected A10 cells

**Fig. 4:**
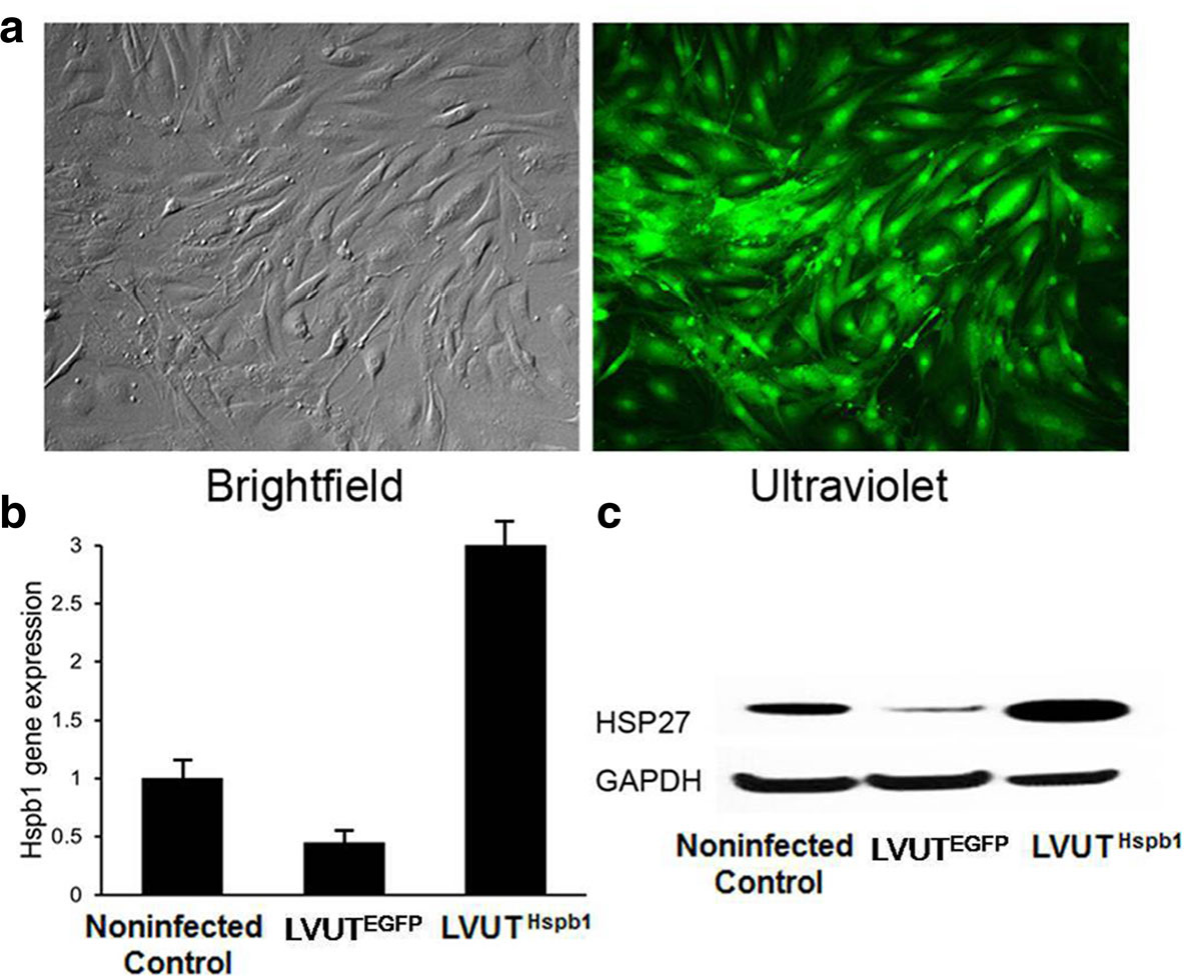
Expressions of Hspb1 and HSP27 in A10 cells. **a –** Lentiviral infection is effective for overexpressing HSP27 in A10 cells, as shown by transfection with the vector containing the green fluorescent protein (GFP) gene, which makes the cells glow green under ultraviolet light. **b –** Quantitative PCR showing the Hspb1 expression level of non-infected A10 cells (non-infected control), A10 cells infected with Lenti6.3-EGFP empty vector (LVUT^EGFP^), and A10 cells infected with LVUT^Hspb1^ (LVUT^Hspb1^). p < 0.05 between each pair of groups. Non-infected control, *n* = 4; LVUTE^GFP^, n = 4; LVUT^Hspb1^, *n* = 4. **c –** A10 cells infected with LVUT^Hspb1^ express higher HSP27 protein levels than LVUT^EGFP^ and non-infected A10 cells. Glyceraldehyde 3-phosphate dehydrogenase (GAPDH) was used as loading control protein

Rat aortic A10 VSMCs were used to study the potential role of HSP27 in the pathogenesis of TAD. A10 cells were efficiently infected with lentiviral particles without affecting the viability of the cells. Both LVUT^EGFP^-infected and LVUT^Hspb1^-infected VSMCs expressed EGFP. Quantitative PCR showed that LVUT^EGFP^ infection decreased *Hspb1* gene expression in A10 cells, but the cells infected with LVUT^Hspb1^ successfully overexpressed *Hspb1*. Western blot showed overexpression of HSP27 in the cells infected with LVUT^Hspb1^ compared with the controls (Fig. 4).

### Overexpression of HSP27 attenuates H_2_O_2_-induced inhibition of cell proliferation

**Fig. 5:**
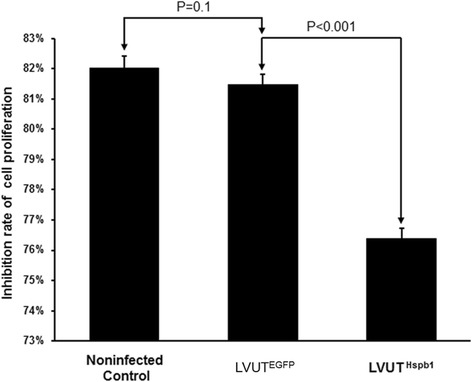
A10 cells were treated with 2 mmol/l of H_2_O_2_ for 2 h. Cell proliferation was assayed with an MTT assay kit. H_2_O_2_ inhibited cell proliferation dramatically, but the inhibition rate was lower in A10 cells infected with LVUT^Hspb1^ than in A10 cells infected with LVUT^EGFP^ (*p* < 0.001). There were no significant differences between the non-infected control and LVUT^EGFP^ (*n* = 3)

Cell proliferation was inhibited after treatment with H_2_O_2_. The inhibition rate had decreased in A10 cells infected with LVUT^Hspb1^ compared with the controls, indicating that overexpression of HSP27 attenuated the H_2_O_2_-induced inhibition of cell proliferation. There were no difference between LVUT^EGFP^ and the non-infected control group (Fig. 5).

### Overexpression of HSP27 attenuates H_2_O_2_-induced cell apoptosis

**Fig. 6:**
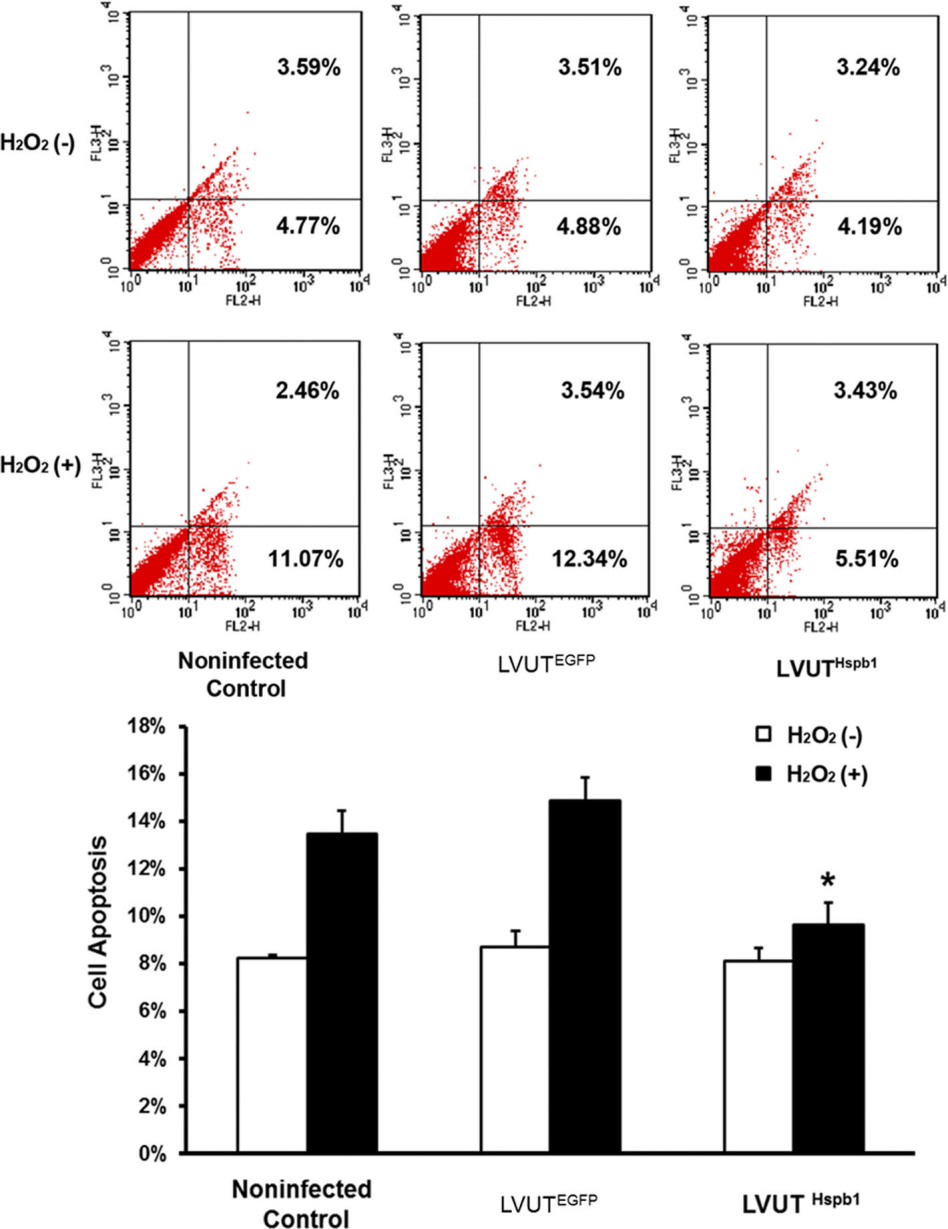
A10 cells were treated with 800 μmol/l of H_2_O_2_ for 24 h. Cell apoptosis was assayed using an Annexin V-PE/7-AAD Apoptosis Detection Kit, and detected using FACS. In the dot plots, the lower left quadrant indicates a live cell population, the lower right quadrant indicates an early apoptotic cell population, and the upper right quadrant indicates a late apoptotic or dead cell population. H_2_O_2_-induced cell apoptosis was significantly attenuated in A10 cells infected with LVUT^Hspb1^ compared with A10 cells infected with LVUT^EGFP^ (*p < 0.001). The rate of apoptosis showed no significant difference between the non-infected control and LVUT^EGFP^ (n = 3)

H_2_O_2_-induced apoptosis of A10 cells was analyzed by FACS. Overexpression of HSP27 significantly reduced the apoptosis in A10 cells infected with LVUT^Hspb1^ compared with the controls. There were no difference between LVUT^EGFP^ and the non-infected control group (Fig. 6).

### Overexpression of HSP27 attenuates H_2_O_2_-induced oxidative stress

**Fig. 7:**
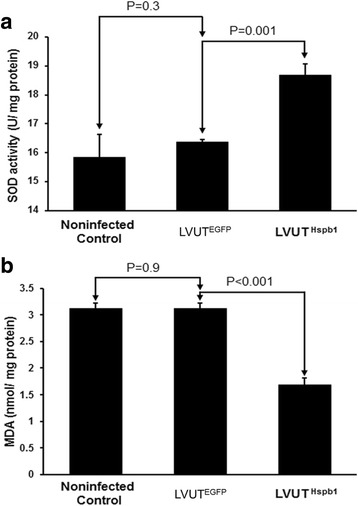
A10 cells were treated with 2 mmol/l of H_2_O_2_ for 2 h. Superoxide dismutase (SOD) activity and lipid peroxidation were tested to reflect the level of oxidative stress. Overexpression of HSP27 significantly increased SOD activity (**a**) and decreased the malondialdehyde level (**b**) in A10 cells infected with LVUT^Hspb1^ compared with A10 cells infected with LVUT^EGFP^. These two indexes showed no significant difference between non-infected controls and LVUT^EGFP^ (n = 3)

To determine the H_2_O_2_-induced oxidative stress level of A10 cells, SOD activity and malondialdehyde levels were measured. Overexpression of HSP27 significantly increased the SOD activity and decreased malondialdehyde levels in A10 cells infected with LVUT^Hspb1^ compared with those infected with LVUT^EGFP^. There was no difference between the LVUT^EGFP^ group and the non-infected controls (Fig. 7).

## Discussion

In our previous proteomic study of the aortic media of patients with TAD, we found that HSP27 was differentially expressed in patients with TAD [10], suggesting that it may play a role in the pathogenesis or progression of TAD.

This study aimed to compare oxidative stress and HSP27 expression in the aortic tissue of patients with TAD and controls, and to explore the potential role of HSP27 in the pathogenesis of TAD using an in vitro model of oxidative stress in VSMCs. Results showed that compared with the controls, elevated oxidative stress was detected in TAD samples. HSP27 expression in aortic tissue samples from patients with TAD had significantly increased, and the protein was mainly produced by VSMCs in the aortic media. We also found that overexpression of HSP27 significantly attenuated H_2_O_2_-induced inhibition of cell proliferation, inhibited cell apoptosis, and decreased the oxidative stress level in VSMCs challenged with H_2_O_2_. Thus, we estimate that HSP27 may play a protective role in oxidative stress-threatened aortas.

Increased oxidative stress may have a destructive role in the vascular system. Oxidative stress is one of the main determinants of pathological remodeling of the arterial wall [14]. Patients with abdominal aortic aneurysm (AAA) show significantly decreased plasma levels of antioxidants and increased specific markers of lipid peroxidation, suggesting that oxidative stress play a key role in the pathogenesis of AAA [15]. It was reported that the endothelial cells and SMCs from AAA have increased oxidative stress, which correlates with telomere attrition [16]. The data reported in this paper directly address the problem of aortic tissue ‘quality’ in patients with dilatation of the proximal aorta, linking wall stress to oxidative injury and VSMC activation.

HSP27 is involved in cellular protection in response to a variety of stresses, including heat shock, toxic compounds and oxidative stress [17]. It is a ubiquitous molecular chaperone that has several other potentially important roles in cell biology, such as cell apoptosis, endothelial barrier function, and inflammation modulation [9, 17–19]. It plays a key role in the arrangement of microstructure of actin, and its phosphorylation increases the dynamics of actin assembly, which is necessary for VSMC migration [20]. HSP27 phosphorylation and its interaction with actin–myosin also play an important role in VSMC contraction [21]. All of these functions may have an impact on the process of atherogenesis, and indeed HSP27 is expressed within arterial lesions, as shown here and in earlier studies [9, 18, 19].

Antibodies against HSP27 have been detected in patients with cancer and coronary diseases, although the antibody levels in patients with coronary diseases were not associated with known coronary risk factors [19]. Desai et al. reported that HSP27 is associated with Hic-5 and regulates the ROS-generating enzyme NADPH oxidase 4, thus plays an important role in myofibroblast differentiation and senescence [11]. This led us to consider the role of HSP27 in VSMCs, the most important component of the medial layer of the aortic wall. Despite using different systems, both our study and that of Desai et al. show that HSP27 plays an important role in oxidative stress-challenged tissues and cells, and that its relationship with NADPH oxidase as a critical oxidant producer in smooth muscle cell physiology is worth further exploration.

FACS was used to analyze oxidative stress-induced cell apoptosis. There were no significant differences in cell apoptosis between the non-infected controls, empty vector control, and HSP27 overexpression groups without H_2_O_2_ treatment. However, after H_2_O_2_ treatment, cell apoptosis was significantly increased in non-infected controls and empty vector controls, and HSP27 overexpression significantly attenuated H_2_O_2_-induced apoptosis. These results indicate that HSP27 plays a protective role in VSMCs in the setting of oxidative stress by attenuating oxidative stress-induced cell apoptosis. These results are important because VSMC is the main cell type in the aortic media and because VSMC apoptosis and depletion are common features of aortic aneurysms and dissections [22].

It was reported that HSP27 plays a role in actin filament remodeling during VSMC migration and contractions [18], and that HSP27 increases the resistance of VSMCs against oxidative stress-induced actin fragmentation [23]. These results are supported by the results obtained from the surgical specimens. Indeed, HSP27 levels were significantly increased in the aortic wall of patients with TAD compared with the controls, and HSP27 was mainly produced by VSMCs in the medial layer of the aortic wall. However, HSP27-negative bands were observed along the tear in the aortic media of TAD patients, suggesting a failure to produce HSP27 along the torn layer. This would make the VSMCs in the torn area less resistant to oxidative stress and more prone to apoptosis.

SOD is a family of reactive oxygen-catalyzing metalloenzymes and includes Mn-SOD, Cu/Zn-SOD, and EC-SOD [24]. SODs are known to play important roles in acute inflammation and to regulate the expression of matrix metalloproteinases (MMPs), a family of enzymes involved in the degradation of elastin and structural collagen [25]. SODs are important antioxidative enzymes in the vessel walls. They represent an important enzymatic antioxidant defense system. Normal activity of Cu/Zn-SOD or EC-SOD or both is necessary to limit increases in superoxide, allowing the release of nitric oxide from the endothelium and normal endothelium-dependent relaxation [26]. Decreased EC-SOD expression has been found to be associated with increased vascular oxidative stress. In this study, total SOD and Cu/Zn-SOD activities were markedly decreased in TAD samples compared with the controls, suggesting an impaired antioxidative mechanism in the aortic wall of patients with TAD.

Malondialdehyde is an end product of lipid peroxidation and is a marker of oxidative stress [27–29]. Malondialdehyde levels were significantly elevated in TAD tissue specimens, suggesting that oxidative stress was significantly increased in the aortas of patients with TAD.

Impaired antioxidative mechanisms and increased oxidative stress of the aortic wall may participate in the pathogenesis of TAD by promoting cell apoptosis and ultimately aggravating aortic destruction. Additional studies are necessary to address this issue. This study was not designed to explain potential cause-and-effect relationships, and the altered oxidative status observed in TAD tissues could also be a consequence of aortic destruction that had occurred earlier.

This study is not without limitations. The aortic samples were obtained during surgical repair, which is performed for end-stage disease, introducing a selection bias and resulting in a type II error because of the great differences between patients with TAD and the controls. The cause-and-effect relationship between HSP27 and TAD progression cannot be determined. This study does not demonstrate a direct relationship between HSP27 and TAD. When dissection takes place, the aortic tissue and vascular cells are challenged with injury and stress. The upregulation of HSP27 may also therefore be a cellular response to the vascular injury. Only HSP27 was examined, and other proteins should be assessed to provide a more comprehensive analysis of protein expression in TAD. Furthermore, the use of H_2_O_2_ results in a mild model of oxidative stress, but it may reflect oxidative stress close to that encountered under physiological conditions compared with other models [30]. Finally, HSPs play a number of different roles in cells during tissue injury and healing [31]. An animal model with differential expression of HSP27 is required to determine the role of HSP27 in TAD and should provide better responses than the A10 cell line.

## Conclusions

We found that oxidative stress was significantly elevated and that the HSP27 level was significantly higher in the aortic media of patients with TAD. HSP27 might play a protective role in the pathogenesis of TAD by attenuating oxidative stress, but additional causative studies are needed to address this point. Understanding the potential role of HSP27 in the pathogenesis of TAD may help to provide potential ways for the prevention and treatment of this severe aortic disease.

## Abbreviations

AAA: abdominal aortic aneurysm
HSP27: Heat shock protein 27
SMC: Smooth muscle cell
TAD: Thoracic aortic dissection
VSMCs: Vascular smooth muscle cells
